# Digital Health Platforms for Breast Cancer Care: A Scoping Review

**DOI:** 10.3390/jcm13071937

**Published:** 2024-03-27

**Authors:** Elayna P. Kirsch, Sameer A. Kunte, Kevin A. Wu, Samantha Kaplan, E. Shelley Hwang, Jennifer K. Plichta, Shivanand P. Lad

**Affiliations:** 1 Department of Neurosurgery, Duke University Medical Center, Durham, NC 27710, USA; 2 Medical Center Library & Archives, Duke University School of Medicine, Durham, NC 27710, USA; 3 Department of Surgery, Duke University Medical Center, Durham, NC 27710, USAjennifer.plichta@duke.edu (J.K.P.)

**Keywords:** breast cancer, digital health, mobile health

## Abstract

Breast cancer is a significant global health concern affecting millions of women each year. Digital health platforms are an easily accessible intervention that can improve patient care, though their efficacy in breast cancer care is unknown. This scoping review aims to provide an overview of existing research on the utilization of digital health platforms for breast cancer care and identify key trends and gaps in the literature. A comprehensive literature search was conducted across electronic databases, including Ovid MEDLINE, Elsevier EMBASE, and Elsevier Scopus databases. The search strategy incorporated keywords related to “digital health platforms”, “breast cancer care”, and associated terminologies. After screening for eligibility, a total of 25 articles were included in this scoping review. The identified studies comprised mobile applications and web-based interventions. These platforms demonstrated various functionalities, including patient education, symptom monitoring, treatment adherence, and psychosocial support. The findings indicate the potential of digital health platforms in improving breast cancer care and patients’ overall experiences. The positive impact on patient outcomes, including improved quality of life and reduced psychological distress, underscores the importance of incorporating digital health solutions into breast cancer management. Additional research is necessary to validate the effectiveness of these platforms in diverse patient populations and assess their impact on healthcare-resource utilization.

## 1. Introduction

Breast cancer remains the most prevalent and deadly malignancy in women worldwide with 2.26 million new cases and 684,000 deaths in 2020 [1]. The projected global burden is expected to be at 28.4 million cases in 2040 with 1 million deaths occurring annually [2,3]. This growing burden is expected to disproportionately impact less economically developed nations, necessitating the need for more affordable solutions to facilitate patient care [4].

The primary treatment options for breast cancer include surgery, chemotherapy, targeted therapy, endocrine therapy, and radiation [5]. The heterogeneity of breast cancers based on tumor burden, receptor expression, and genomic variability necessitates the coordination of multidisciplinary teams to personalize treatment to a patient’s disease. Receiving care from multiple teams in complex healthcare systems can be challenging for patients to navigate [6].

Patients undergoing treatment for breast cancer have high rates of emotional distress—studies show that at least 38% of patients suffer from depression and 32% of patients report anxiety [7,8]. While rates of emotional distress have been decreasing, likely due to improved psychosocial interventions, it is critical to find other methods of managing the psychological burden of breast cancer [9]. One potential intervention for reducing emotional distress in breast cancer patients is to augment patient education and engagement in their healthcare journey. Patients who are well informed and involved in decision making have lower depression scores and higher quality-of-life metrics [10].

Digital health is a relatively new mode of patient education and engagement that can help to disseminate evidence-based information, help guide decision making, and bolster self-efficacy [11,12]. These interventions are highly accessible to patients, as 70–80% of the world’s population owns a smartphone, and the cost of digital health platforms is often negligible to users [13]. There is a preponderance of digital health tools aimed at patient education in topics ranging from diabetes, electrophysiology, maternal health, and rheumatological disease [14,15,16,17]. The quality of digital health tools, however, is highly variable, with little guidance or oversight over these interventions [18]. The goal of this scoping review is to summarize the current literature on digital health interventions available for patients undergoing treatment for breast cancer, report their potential benefits to patient care, and identify new opportunities and applications in this space.

## 2. Materials and Methods

**Figure 1:**
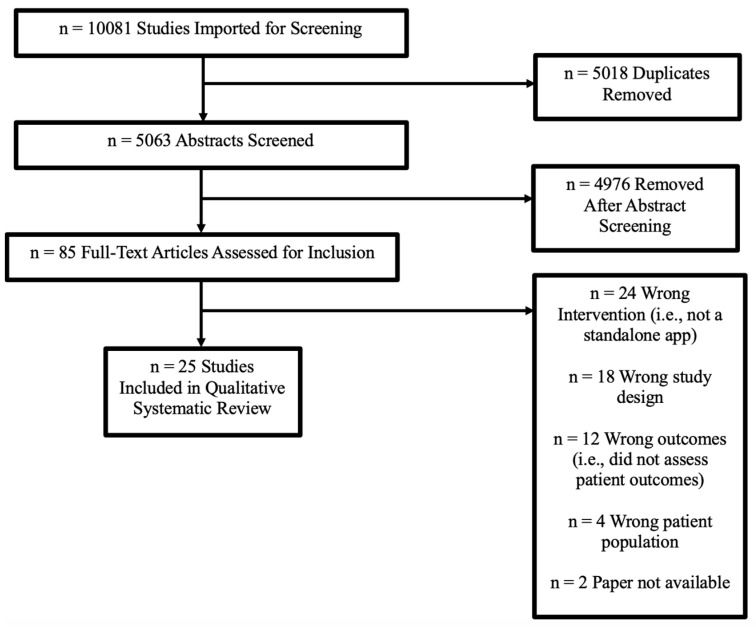
Flow diagram of study selection.

A literature search was conducted through Ovid MEDLINE, Elsevier EMBASE, and Elsevier Scopus databases on 14 March 2022, using the search strategies listed in Supplementary Tables S1–S3. Covidence was used for screening abstracts and full texts prior to data extraction. We screened a total of 5063 abstracts following PRIMSA guidelines. After a full-text review by two independent authors (EK and SK), 25 studies were included. We did not include review articles in the final extraction, as the intention was to identify primary studies, but we did review the references for additional citations, which did not yield new studies. The flow diagram of study selection is shown in Figure 1. Data were extracted from the final list of included studies. The two reviewers independently charted data and reviewed findings to come to a consensus about the study findings.

We included any studies describing the use of digital tools, specifically smartphone or web-based applications, in improving outcomes for breast cancer patients. We excluded strictly provider-facing tools, electronic healthcare messaging platforms, studies tracking website use, general health or fitness trackers, development and feasibility studies that did not report patient outcomes, or studies that did not pertain to the management of breast cancer.

Assessment of bias and quality was completed for all included studies. The Methodological Index for Nonrandomized Studies (MINORS) criteria was used for all cohort studies. MINORS consists of an 8-item checklist for non-comparative studies with each item being scored 0 (not reported), 1 (inadequately reported), or 2 (adequately reported). Overall scores range from 0–16. The Joanna Briggs Institute critical appraisal checklist was used to appraise bias for randomized control trials. The checklist contains 10 times, each scored as “Yes”, “No”, “NA” (not applicable), or “Not reported”.

## 3. Results

### 3.1. Cohort Studies

Five cohort studies were identified describing the development, feasibility, and implementation of digital health platforms in the care of breast cancer patients. The risk of bias as determined by the MINORS criteria was low, as all included studies scored at least 13 of 16 possible points (Table 1). Buscemi et al. piloted a smartphone application called My Guide, an application specifically designed for Hispanic breast cancer survivors (Table 2) [19]. The researchers found that both patient engagement and satisfaction were high after using the application. There was also a significant improvement in breast cancer knowledge after use of the application, but no difference in quality of life. Yu et al. report that, in a cohort of 4475 breast cancer patients undergoing multidisciplinary treatment (i.e., chemotherapy and radiation), the use of a smartphone application was associated with increased adherence to therapy [20]. A feasibility study by Ponder et al. investigated patient satisfaction and patient-reported outcomes after using a smartphone application called ManageMySurgery (MMS) [21]. MMS is an educational tool designed to help patients navigate the perioperative environment. The majority of the 33 study participants undergoing either a mastectomy or lumpectomy found the application useful, and there was a significant decrease in anxiety and depression after application use. Lin et al. studied a decision support aid for 11 women considering breast reconstruction surgery in Taiwan [22]. The application, called Pink Journey, provided information on various treatment options, encouraged patients to explore their values, and presented the options within the context of patients’ concerns. The app was found to help reduce decisional conflict, and the majority of study participants found the application useful. Lastly, Wyatt et al. studied the use of a web-based decision aid in 225 women newly diagnosed with breast cancer; the majority of patients found the application helpful and easy to use, and there was an increase in decision-making confidence, particularly among patients with low baseline confidence [23].

### 3.2. Randomized Control Trials (RCT)

#### 3.2.1. Web-Based Platforms

There were six RCTs on web-based education tools. The risk of bias of web-based RCTs was low as determined by the Joanna Briggs Institute critical appraisal tool (Table 3). The item “Were participants blind to treatment assignment?” was deemed “NA” (not applicable) for all studies as blinding participants to the use of a digital health intervention is unfeasible. Admiraal et al. described the use of a web-based psychoeducational program, ENCOURAGE, in patients diagnosed with primary breast cancer and undergoing chemotherapy (Table 4) [24]. A total of 138 patients were enrolled in the RCT. While there was no difference between the subjects who used the web-based platform and those who did not, all groups reported an improvement in optimism, control, distress, and quality of life. Interestingly, patients who were clinically distressed at baseline had a greater improvement in optimism and control in the intervention group. Three studies described the use of the Comprehensive Health Enhancement Support System (CHESS), a web-based resource that integrates information, support, decision, and analysis tools for women recently diagnosed with breast cancer. An RCT of 257 patients found that patients who used CHESS had better social support, quality of life, and healthcare competence than patients with and without access to the internet [25]. In another RCT of 60 patients, CHESS increased participation in care among minority women and women with a lower formal education status, while also improving the wellbeing of women without private health insurance [26]. Kim et al. studied the use of CHESS along with a trained mentor who provided additional cancer information [27]. CHESS alone improved information competence, but the addition of a mentor improved emotional–social competence and emotional functioning. Ventura et al. conducted an RCT of 226 women with early-stage breast cancer scheduled for surgery and found no significant difference in anxiety and depression levels among patients who used a computer-based educational program [28]. On the contrary, Korkmaz et al. report the use of a web-based education platform for patients undergoing breast surgery with axillary lymph node dissection led to decreased anxiety and improved quality of life [29].

Five studies described the use of a web-based decision aid (Table 4). Manne et al. found that the use of B-Sure, an interactive decision aid that provided information on contralateral prophylactic mastectomy (CPM) and documented patient experiences, was associated with greater knowledge and increased clarity [30]. There were no differences in self-efficacy, perceived risk, worry, or motivations for proceeding with surgery. Another web-based decision aid regarding breast reconstruction after mastectomy, named BRAID, was studied in 55 participants. While knowledge level, satisfaction, preparation, and decisional conflict improved in all patients enrolled, there was no statistical difference between the control and BRAID groups [31]. Politi et al. investigated the use of BREASTChoice, also a web-based decision aid for breast reconstruction after mastectomy [32]. A total of 120 patients participated in the study; the intervention group had greater knowledge and confidence about reconstruction, but there were no differences in decisional conflict or quality of life between groups. Two studies explored BRECONDA, a web-based decision aid intended to help women with a hereditary risk of breast cancer make decisions regarding risk-reducing mastectomy. Both RCTs reported that the use of BRECONDA was associated with a decrease in decisional conflict, an increase in knowledge, and satisfaction with the information [33,34].

#### 3.2.2. Smartphone-Application-Based Platforms

There were a small number of studies that focused on symptom monitoring and treatment adherence. The risk of bias of smartphone-application-based RCTs was low, as determined by the Joanna Briggs Institute critical appraisal tool (Table 5). The item “Were participants blind to treatment assignment?” was deemed “NA” (Not applicable) for all studies, as blinding participants to the use of a digital health intervention is unfeasible Zhu et al. studied a smartphone application developed in China, named Breast Cancer e-Support (BCS) (Table 6) [35]. BCS improved self-efficacy, symptoms, and quality of life in women with breast cancer three months after starting chemotherapy. A study based in Sweden on women undergoing neoadjuvant chemotherapy found that the use of a smartphone application, called Interkator, reduced physical symptoms and symptom distress, and improved emotional function. Interkator allowed patients to log their symptoms, communicate with healthcare professionals, and access educational resources about chemotherapy side effects [36]. A third study investigating a symptom-monitoring smartphone application, Msymptom, reported the application was associated with lower physical symptom scores and nausea/vomiting scores. Interestingly, patients in the control group had significantly higher sexual function and pleasure [37]. Lastly, a group based in Taiwan conducted an RCT on 112 women with recently diagnosed nonmetastatic breast cancer, randomized to either the breast cancer self-management support (BCSMS) mHealth mobile application or usual care. The mobile application group had higher quality-of-life scores three months after being introduced to the intervention [38].

Baik et al. completed an RCT among 80 Latina breast cancer survivors [39]. Participants were randomized between two applications: My Guide, aimed to improve quality of life and reduce symptoms, and My Health, aimed to promote healthy lifestyle behaviors. There was an improvement in overall wellbeing for low application users (less than 60 min per week) of My Guide, but increased social wellbeing among high application users of My Health. Yanez et al. also described the use of My Guide and My Health, but specifically studied symptom burden and quality of life [40]. Use of both My Guide and My Health were associated with decreased symptom burden and improved quality of life. Oswald et al. conducted a secondary analysis comparing My Guide and My Health in 78 Latina breast cancer survivors; patients using My Guide had a greater improvement in knowledge and a reduction in self-blame compared to those who used My Health [41].

Fang et al. conducted an RCT using the smartphone application Pink Journey, which was previously discussed [42]. Ninety-six women considering breast reconstruction were enrolled in the RCT. There were no differences in primary outcomes, including decisional conflict, regret, anxiety, or depression; however, there was a decrease in body-image distress for the intervention group compared to the controls. Foley et al. developed a smartphone application in Ireland to help deliver information to breast cancer patients in the perioperative period [43]. A total of 39 patients were enrolled in the RCT, which found that anxiety and depression were lower in the control group than in the group exposed to the application.

**Table 5:** Risk of bias and quality assessment for smartphone-application-based randomized control trials.

Joanna Briggs Institute Critical Appraisal	Baik 2020 [39]	Fang 2021 [42]	Fjell 2020 [36]	Foley 2016 [43]	Hou 2020 [38]	Oswald 2021 [41]	Ozturk 2021 [37]	Yanez 2019 [40]	Zhu 2018 [35]
Was true randomization used for assignment of participants to treatment groups?	Yes	Yes	Yes	Yes	Yes	Yes	Yes	Yes	Yes
Was allocation to treatment groups concealed?	Yes	Yes	Yes	Yes	Yes	Yes	Yes	Yes	Yes
Were treatment groups similar at the baseline?	Yes	Yes	Yes	Yes	Yes	Yes	Yes	Yes	Yes
Were participants blind to treatment assignment?	NA	NA	NA	NA	NA	NA	NA	NA	NA
Were those delivering treatment blind to treatment assignment?	Not reported	No	No	Not reported	No	Not reported	Not reported	Not reported	Yes
Were outcomes assessors blind to treatment assignment?	Yes	Yes	Yes	Yes	Yes	Yes	Yes	Yes	Yes
Were treatment groups treated identically other than the intervention of interest?	Yes	Yes	Yes	Yes	Yes	Yes	Yes	Yes	Yes
Was follow up complete and if not, were differences between groups in terms of their follow up adequately described and analyzed?	Yes	Yes	Yes	Yes	Yes	Yes	Yes	Yes	Yes
Were participants analyzed in the groups to which they were randomized?	Yes	Yes	Yes	Yes	Yes	Yes	Yes	Yes	Yes
Were outcomes measured in the same way for treatment groups?	Yes	Yes	Yes	Yes	Yes	Yes	Yes	Yes	Yes
Were outcomes measured in a reliable way?	Yes	Yes	Yes	Yes	Yes	Yes	Yes	Yes	Yes
Was appropriate statistical analysis used?	Yes	Yes	Yes	Yes	Yes	Yes	Yes	Yes	Yes
Was the trial design appropriate, and any deviations from the standard RCT design accounted for in the conduct and analysis of the trial?	Yes	Yes	Yes	Yes	Yes	Yes	Yes	Yes	Yes

**Table 6:** Randomized control trials using smartphone-application-based platforms.

Paper	Title	Intervention	Country	Number of Subjects	Key Outcomes
Baik 2020 [39]	Patterns of use of smartphone-based interventions among latina breast cancer survivors: Secondary analysis of a pilot randomized controlled trial	*My Guide* and *My Health* apps	USA	80	MyGuide app was associated with improved well-being in participants with low use MyHealth app was associated with increased social well-being in participants with high use
Fang 2021 [42]	Long-Term Effectiveness of a Decision Support App (Pink Journey) for Women Considering Breast Reconstruction Surgery: Pilot Randomized Controlled Trial	*Pink Journey* app	Taiwan	96	Application resulted in decreased body image distress compared to control
Fjell 2020 [36]	Reduced symptom burden with the support of an interactive app during neoadjuvant chemotherapy for breast cancer e A randomized controlled trial	*Interaktor* app	Sweden	150	Application reduced symptom burden, symptom distress, and improved emotional function
Foley 2016 [43]	PATI: Patient accessed tailored information: A pilot study to evaluate the effect on preoperative breast cancer patients of information delivered via a mobile application	unnamed app	Ireland	39	Control was associated with lower anxiety and depression than the application
Hou 2020 [38]	Quality of Life of Women After a First Diagnosis of Breast Cancer Using a Self-Management Support mHealth App in Taiwan: Randomized Controlled Trial	*BCSMS* app	Taiwan	112	Application increased QoL scores
Oswald 2021 [41]	Effects of smartphone interventions on cancer knowledge and coping among Latina breast cancer survivors: Secondary analysis of a pilot randomized controlled trial	*MyGuide* and *MyHealth* apps	USA	78	MyGuide app resulted in greater improvements to knowledge and reduction in self-blame than MyHealth app
Ozturk 2021 [37]	The Effect of the Mobile Application-Based Symptom Monitoring Process on the Symptom Control and Quality of Life in Breast Cancer Patients	*Msemptom* app	Turkey	57	Application associated with lower physical symptom scores and nausea/vomiting scores
Yanez 2019 [40]	Brief culturally informed smartphone interventions decrease breast cancer symptom burden among Latina breast cancer survivors	*MyGuide* and *MyHealth* apps	USA	80	MyGuide and MyHealth apps were associated with decreased symptom burden and improved QoL
Zhu 2018 [35]	Mobile Breast Cancer e-Support Program for Chinese Women With Breast Cancer Undergoing Chemotherapy (Part 2): Multicenter Randomized Controlled Trial	*BCS* app	China	114	Application improved self-efficacy, symptom burden, and QoL

## 4. Discussion

This review identified numerous studies discussing many of the digital health platforms available for patients undergoing breast cancer treatment. The majority of studies suggest that digital health platforms can enhance the management of breast cancer and improve the quality of life for patients. Digital health platforms have been shown to improve patient outcomes and quality of life by increasing patient education, encouraging patient involvement, and reducing illness anxiety [44,45]. Given the prevalence of breast cancer worldwide, digital health platforms have the potential to make a positive impact on a significant number of patients.

Importantly, a handful of studies identified in this review suggest that digital health platforms may be most useful in patients who are more distressed at baseline, are of minority status, have lower levels of education, or lack insurance [24,26]. Social determinants of health are known to be associated with morbidity and mortality in breast cancer patients. Goel et al. report that a lower neighborhood socioeconomic status is associated with a shorter breast cancer-specific survival, even after controlling for individual sociodemographic, access to care, comorbidities, tumor characteristics, and the National Comprehensive Cancer Network (NCCN) treatment paradigms [46]. Furthermore, non-Hispanic Black patients and Hispanic patients are more likely to undergo mastectomies and receive delayed treatment than non-Hispanic White patients [47]. Digital health platforms can provide important education to patients who may have difficulty accessing care otherwise.

Studies identified in this review also suggest digital health platforms improve the overall symptom burden from breast cancer treatment. One study specifically found that treatment adherence was improved by patients using their digital health platform, an association likely mediated by a decrease in symptom burden [20]. The oncologic literature suggests patients who routinely report symptoms through digital health platforms have a lower symptom burden due to the increased awareness of serious adverse reactions and earlier detection by patients and providers [48]. Furthermore, by providing education to patients, expectations are more easily managed, and there is a reduction in patient visits to emergency departments [49].

While the majority of studies ascertained in this review revolve around adjuvant treatment for breast cancer, a handful specifically targeted patients in the perioperative period. Digital health platforms that were designed as decision-making aids were overall shown to improve education and reduce decisional conflicts among patients [23,30,32,33,34]. However, one study reported that the use of a digital health platform as an educational tool for patients prior to surgery increased anxiety and depression scores [43]. This could potentially be explained by the idea of information overload. A surplus of information is seen as overwhelming to patients, and conflicting information regarding treatment options leads to difficulty in decision making [50]. Nonetheless, material on digital health platforms should be curated by expert physicians to ensure accurate and relevant information, and providers should be prepared to discuss information acquired through digital health platforms and the broader web with their patients.

The efficacy of digital health platforms in patients from resource-limited backgrounds suggests that this intervention will be invaluable in addressing the disproportionate increase in breast cancer cases that resource-limited nations are facing [4]. Digital health is highly accessible given the ubiquity of smartphones as well as the minimal cost of digital health applications to users [13]. Expanding access to digital health globally requires further consideration than simply increasing patient access to technology. Support will be required from governmental and private industry groups to help develop reliable access to electricity and the Internet [51]. Additionally, technological illiteracy poses one of the largest barriers preventing digital health uptake in low-income countries [52]. Institutional-level interventions will be needed to address these barriers to aid in the growth of digital health. Finally, it is crucial that digital health platforms integrate culturally relevant information for the populations they seek to serve [53]. Considering local values and customs in the creation of digital health interventions will help to better serve patients globally.

The primary limitations of this study are common to scoping reviews. A meta-analysis or study appraisal was not conducted, given the differences in intervention type and patient populations across the studies identified. Nevertheless, this scoping review was conducted through a rigorous and standardized process. A limitation to investigating the efficacy of digital health platforms is the lack of a formal regulatory process overseeing the development of such tools. However, the majority of studies had institutional protocols for the content validity of the digital health platform. Lastly, it is difficult to extrapolate the results of each study to all patients undergoing breast cancer treatment, given the differences in demographics and stage of cancer diagnosis between the study populations.

## 5. Conclusions

We present a scoping review analyzing the landscape of digital health platforms for patients undergoing breast cancer treatment. There are a select number of web-based and smartphone digital health tools that have been developed globally to assist patients with breast cancer. Overall, digital health platforms aid in patient education, are associated with higher quality of life, lower levels of anxiety and depression, a decrease in overall symptom burden, and can assist in important decision-making regarding treatment options. Further research should validate the use of digital health platforms in a broader patient population and investigate the impact of digital health on healthcare-resource utilization in breast cancer management.

## Figures and Tables

**Table 1:** Risk of bias and quality assessment for cohort studies.

MINORS	Buscemi 2019 [19]	Lin 2021 [22]	Ponder 2021 [21]	Wyatt 2017 [23]	Yu 2021 [20]
A clearly stated aim	2	2	2	2	2
Inclusion of consecutive patients	2	2	2	2	2
Prospective collection of data	2	2	2	2	2
Endpoints appropriate to the aim of the study	2	2	2	2	2
Unbiased assessment of study endpoints	2	2	2	2	2
Follow-up period appropriate to the aim of the study	2	1	2	2	2
Loss to follow up less than 5%	0	2	2	0	0
Prospective calculation of study size	1	1	1	1	1
**Total**	13	14	15	13	13

**Table 2:** Cohort studies for digital health interventions.

Paper	Title	Intervention	Country	Number of Subjects	Key Outcomes
Buscemi 2019 [19]	Feasibility of a Smartphone-based pilot intervention for Hispanic breast cancer survivors: a brief report	*My Guide* app	USA	25	Application had high patient engagement and satisfaction Application significantly improved breast cancer knowledge
Lin 2021 [22]	Development and Usability Testing of a Decision Support App for Women Considering Breast Reconstruction Surgery	*Pink Journey* app	Taiwan	11	Majority found the application useful Application reduced decision conflict
Ponder 2021 [21]	Mobile Health Application for Patients Undergoing Breast Cancer Surgery: Feasibility Study	*MMS* app	USA	33	Majority found the application useful Application associated with significant decrease in anxiety and depression
Wyatt 2017 [23]	A personalized, web-based breast cancer decision making application: a pre-post survey	unnamed app	USA	255	Majority found the application helpful and easy to use Application associated with an increase in decision making confidence
Yu 2021 [20]	A Smartphone-Based App to Improve Adjuvant Treatment Adherence to Multidisciplinary Decisions in Patients With Early-Stage Breast Cancer: Observational Study	*full course management system* app	China	4475	Application associated with increased therapy adherence

**Table 3:** Risk of bias and quality assessment for web-based randomized control trials.

Joanna Briggs Institute Critical Appraisal	Admiraal 2017 [24]	Gustafson 2008 [25]	Gustafson 2001 [26]	Kim 2020 [27]	Korkmanz 2020 [29]	Manne 2020 [30]	Manne 2016 [31]	Politi 2020 [32]	Sherman 2017 [33]	Sherman 2016 [34]	Ventura 2016 [28]
Was true randomization used for assignment of participants to treatment groups?	Yes	Yes	Yes	Yes	Yes	Yes	Yes	Yes	Yes	Yes	Yes
Was allocation to treatment groups concealed?	Yes	Yes	Yes	Yes	Yes	Yes	Yes	Yes	Yes	Yes	Yes
Were treatment groups similar at the baseline?	Yes	Not reported	Yes	Yes	Yes	Not reported	Yes	Yes	Yes	Yes	Yes
Were participants blind to treatment assignment?	NA	NA	NA	NA	NA	NA	NA	NA	NA	NA	NA
Were those delivering treatment blind to treatment assignment?	No	Not reported	Not reported	Not reported	Not reported	No	Not reported	Not reported	Yes	Yes	Not reported
Were outcomes assessors blind to treatment assignment?	Yes	Yes	Yes	Yes	Yes	Yes	Yes	Yes	Yes	Yes	Yes
Were treatment groups treated identically other than the intervention of interest?	No	Yes	Yes	Yes	Yes	Yes	Yes	Yes	Yes	Yes	Yes
Was follow up complete and if not, were differences between groups in terms of their follow up adequately described and analyzed?	Yes	Yes	Yes	Not reported	Yes	Yes	Yes	Yes	Yes	Yes	Yes
Were participants analyzed in the groups to which they were randomized?	Yes	Yes	Yes	Yes	Yes	Yes	Yes	Yes	Yes	Yes	Yes
Were outcomes measured in the same way for treatment groups?	Yes	Yes	Yes	Yes	Yes	Yes	Yes	Yes	Yes	Yes	Yes
Were outcomes measured in a reliable way?	Yes	Yes	Yes	Yes	Yes	Yes	Yes	Yes	Yes	Yes	Yes
Was appropriate statistical analysis used?	Yes	Yes	Yes	Yes	Yes	Yes	Yes	Yes	Yes	Yes	Yes
Was the trial design appropriate, and any deviations from the standard RCT design accounted for in the conduct and analysis of the trial?	Yes	Yes	Yes	Yes	Yes	Yes	Yes	Yes	Yes	Yes	Yes

**Table 4:** Randomized control trials using web-based interventions.

Paper	Title	Intervention	Country	Number of Subjects	Key Outcomes
Admiraal 2017 [24]	Web-Based Tailored Psychoeducation for Breast Cancer Patients at the Onset of the Survivorship Phase: A Multicenter Randomized Controlled Trial	*ENCOURAGE* website	Netherlands	138	No significant difference between website and control on optimism, control, distress, or QoL Website led to greater improvements in optimism in participants who were clinically distressed at baseline
Gustafson 2008 [25]	Internet-Based Interactive Support for Cancer Patients: Are Integrated Systems Better?	*CHESS* website	USA	257	Website group had better social support, QoL, health-care competence
Gustafson 2001 [26]	Effect of Computer Support on Younger Women with Breast Cancer	*CHESS* website	USA	60	Website led to increased participation among minority women and women with low educations status Website improved wellbeing for women without private insurance
Kim 2020 [27]	Understanding how e-health interventions meet psychosocial needs of breast cancer patients: The pathways of influence on quality of life and cancer concerns	*CHESS* website	USA	326	Website alone improved information competence Website with trained cancer information mentor improved emotional-social competence and emotional functioning
Korkmanz 2020 [29]	An Evaluation of the Influence of Web-Based Patient Education on the Anxiety and Life Quality of Patients Who Have Undergone Mammaplasty: a Randomized Controlled Study	*Bilinclihasta* website	Turkey	75	Website resulted in decreased anxiety and increased QoL compared to control
Manne 2019 [30]	B-Sure: a randomized pilot trial of an interactive web-based decision support aid versus usual care in average-risk breast cancer patients considering contralateral prophylactic mastectomy	*B-sure* website	USA	93	Decision aid resulted in greater knowledge and increased clarity
Manne 2015 [31]	Acceptability and pilot efficacy trial of a web-based breast reconstruction decision support aid for women considering mastectomy	*BRAID* website	USA	55	There was no difference between decision aid group and control group in improvements to knowledge, satisfaction, preparation, and decisional conflict
Politi 2020 [32]	A Randomized Controlled Trial Evaluating the BREASTChoice Tool for Personalized Decision Support About Breast Reconstruction After Mastectomy	*BREASTChoice* website	USA	376	Decision aid led to greater knowledge and confidence than the control
Sherman 2017 [33]	Facilitating decision-making in women undergoing genetic testing for hereditary breast cancer: BRECONDA randomized controlled trial results	*BRECONDA* website	Australia	64	Decision aid was associated with decrease in decision conflict and increase in knowledge and satisfaction
Sherman 2016 [34]	Reducing Decisional Conflict and Enhancing Satisfaction with Information among Women Considering Breast Reconstruction following Mastectomy: Results from the BRECONDA Randomized Controlled Trial	*BRECONDA* website	Australia	222	Decision aid was associated with decrease in decision conflict and increase in knowledge and satisfaction
Ventura 2016 [28]	Challenges of evaluating a computer-based educational programme for women diagnosed with early-stage breast cancer: a randomised controlled trial	*SIRI* website	Sweden	226	Intervention had no significant effect on self-efficacy, health participation, anxiety, or depression

## Data Availability

Data is contained within the article or Supplementary Materials.

## Supplementary Materials

The following supporting information can be downloaded at: https://www.mdpi.com/article/10.3390/jcm13071937/s1, Table S1: Search Strategy Report from Ovid Medline. Table S2: Search Strategy Report from Embase. Table S3: Search Strategy Report from Scopus. Table S4: Preferred Reporting Items for Systematic reviews and Meta-Analyses extension for Scoping Reviews (PRISMA-ScR) Checklist. Reference [54] are cited in the supplementary materials.
